# The prospective relationship between social cohesion and depressive symptoms among older adults from Central and Eastern Europe

**DOI:** 10.1136/jech-2018-211063

**Published:** 2018-11-01

**Authors:** Carla Bertossi Urzua, Milagros A Ruiz, Andrzej Pajak, Magdalena Kozela, Ruzena Kubinova, Sofia Malyutina, Anne Peasey, Hynek Pikhart, Michael Marmot, Martin Bobak

**Affiliations:** 1 Research Department of Epidemiology and Public Health, University College London, London, UK; 2 Institute of Public Health, Faculty of Health Sciences, Jagiellonian University Medical College, Krakow, Poland; 3 Centre for Environmental Health Monitoring, National Institute of Public Health, Prague, Czech Republic; 4 Research Institute of Internal and Preventive Medicine, Branch of the Institute of Cytology and Genetics, SB RAS, Novosibirsk, Russia; 5 Novosibirsk State Medical University, Novosibirsk, Russia; 6 UCL Institute of Health Equity and Research Department of Epidemiology and Public Health, University College London, London, UK

**Keywords:** Central and Eastern Europe, cohort study, depressive symptoms, depression, social cohesion, social capital

## Abstract

**Background:**

Social cohesion has a potential protective effect against depression, but evidence for Central and Eastern Europe is lacking. We investigated the prospective association between social cohesion and elevated depressive symptoms in the Czech Republic, Russia and Poland, and assessed whether alcohol drinking and smoking mediated this association.

**Methods:**

Cohort data from 15 438 older urban participants from the Health, Alcohol and Psychosocial factors In Eastern Europe project were analysed. Baseline social cohesion was measured by five questions, and depressive symptoms were measured 3 years later by the 10-item Center for Epidemiological Depression (CES-D) Scale. Nested logistic regression models estimated ORs of elevated depressive symptoms (CES-D 10 score ≥4) by z-scores and tertiles of social cohesion.

**Results:**

Per 1 SD decrease in social cohesion score, adjusted ORs of elevated depressive symptoms were 1.13 (95% CI 1.05 to 1.23) and 1.05 (95% CI 0.99 to 1.13) in men and women, respectively. Further adjustment for smoking and drinking did not attenuate these associations in either men (OR=1.13, 95% CI 1.05 to 1.22) or women (OR=1.05, 95% CI 0.99 to 1.13). Similarly, the fully adjusted ORs comparing the lowest versus highest social cohesion tertile were 1.33 (95% CI 1.10 to 1.62) in men and 1.18 (95% CI 1.01 to 1.39) in women.

**Conclusions:**

Lower levels of social cohesion was associated with heightened depressive symptoms after a 3-year follow-up among older Czech, Russian and Polish adults. These effects appeared stronger in men, and alcohol and smoking played no appreciable role in this association.

## Introduction

Depression is a major public health concern in Central and Eastern Europe (CEE), and strongly predicts cardiovascular and all-cause mortality in this region.^1^ Depression rates in CEE are substantially higher than in other parts of Europe,^2^ potentially reflecting the disruptions of the societal transition after the collapse of communism in the early 1990s.^3^ The accelerated shift towards a market economy resulted not only in dramatic health declines, but also rapid increases in social inequalities and social distress.^4^ In particular, the unprecedented social, economic and political changes have been theorised to undermine the regulatory aspects of social integration, such as social control and norms.^5^ These unparalleled changes disrupted the psychosocial environment, due to the lack of security and collapse of social institutions.^5 6^

Social cohesion is a cultural dimension of the psychosocial environment,^5^ and has been defined as a component of cognitive social capital that is expressed by altruism, reciprocity, values and norms between members of a community.^7 8^ Markers of cognitive social capital, including social cohesion, have become increasingly recognised as determinants of common mental disorders, including depression, according to three reviews. This area of research is relatively well-established, but most evidence is cross-sectional and limited to high-income countries with particular sociocultural settings; none of the reviews included prospective studies from CEE or other lower income countries.^8–10^

The relationship between social cohesion and depression may be particularly important in postcommunist countries. The rapid transition may have not only aggravated poor mental health in the region,^11^ but also social cohesion^12^ which is known to collapse under political and economic crises. Such effects appear long lasting as decades following the transition from communism to democracy, social cohesion levels were substantially lower in CEE than in Northwestern Europe in 2003–2008.^13^

Social cohesion may protect against depressive disorders by discouraging deleterious health behaviours,^14 15^ through the regulation of social norms and protection against daily stressors. The former may modify the social acceptability of these behaviours, while the latter may reduce their likelihood as coping strategies against stress.^5 9^ Although studies have independently shown that low levels of social cohesion predict harmful health behaviours on one hand, and that smoking and alcohol are risk factors for depressive disorders on the other hand, very few studies have tested whether health behaviours may partially explain the relationship between social cohesion and depression. While Berkman and colleagues have reported that behavioural pathways account for only 20% of the relationship between markers of social capital and health more broadly,^5^ the role of alcohol drinking frequency and smoking status as potential mechanisms between social cohesion and depression may be especially important for CEE, given the region’s high burden of substance use disorders.^16 17^

The aim of this paper was to assess the prospective association between social cohesion and elevated depressive symptoms among older adults in three CEE countries, and to explore whether alcohol and smoking mediate this association.

## Methods

### Participants

We used data from the Health, Alcohol and Psychosocial factors In Eastern Europe (HAPIEE) project, a population-based urban cohort study conducted in the Czech Republic (six towns), Russia (Novosibirsk), Poland (Krakow) and Lithuania (Kaunas).^4^ As Lithuania joined HAPIEE at follow-up, we analysed Czech, Russian and Polish participant data. Baseline data collection of adults aged 45–69 years occurred between 2002 and 2005, and recruited 28 945 individuals with response rates ranging from 55% (Czech Republic) to 61% (Russia and Poland). Follow-up data were collected between 2006 and 2008, which successfully re-examined 18 011 participants of the original cohort with response rates of 59% in the Czech Republic, 66% in Russia and 62% in Poland.

### Measurements and variables

Depressive symptoms at both baseline and follow-up were employed for the present analysis, along with study covariates at baseline.

Depressive symptomatology at follow-up was assessed using the 10-item Centre for Epidemiologic Studies Depression (CES-D) Scale, a shortened version comprising 10 of the 20 items included in the original CES-D 20 Scale.^18 19^ The CES-D Scale is designed to measure self-reported depressive symptoms in the general population.^20^ Czech, Russian and Polish language versions of the scale have been found to perform well in each intended country.^21–23^ The CES-D 10 has been found to have excellent screening properties for major depression in older adults, and has sufficiently identified depressive cases as those diagnosed by clinicians.^24^ The CES-D 10 measured whether 10 specific symptoms were experienced ‘for much of the time’ during the past week with yes (1) or no (0) response options. After deriving CES-D scores (ranging from 0 to 10) from these responses, the recommended cut-off was used to classify participants with a score of 4 or higher as having elevated depressive symptoms at follow-up.^24^

Social cohesion was measured using a 5-item scale asking participants: (i) whether they felt safe in their neighbourhoods during the day, (ii) during the night, (iii) whether their neighbours would help them if needed, (iv) whether there was trust among neighbours and (v) whether they trusted their neighbours. Responses were collected on a five-point Likert Scale ranging from 1 (‘never’) to 5 (‘always’). Internal consistency of the scale was considered acceptable according to Cronbach’s alpha (α=0.76). Item responses were summed to generate a score ranging from 5 to 25, whereby higher scores reflected higher levels of social cohesion. Social cohesion scores were analysed using z-scores and grouped by high (21–25), medium (18–20) and low tertiles (5–17).

Alcohol drinking frequency was categorised as never, less than 1 month, one to three times a month, one to four times a week and five or more times a week. Smoking status was classified as never, past and current smoking.

Age, sex, country, marital status, educational level, economic deprivation, self-rated health and depressive symptoms were included as potential confounders at baseline. Economic deprivation, measured as a score from 0 to 12, indicated how often participants lacked enough money for food, clothing or paying bills. Baseline depressive symptoms were measured using the original CES-D 20 Scale; which assessed how frequently 20 depressive symptoms were experienced during the past week on a four-point Likert Scale ranging from ‘less than once a day’ (0), ‘1–2 days’ (1), ‘3–4 days’ (2) to ‘5–7 days’ (3). To account for baseline depressive symptoms, CES-D 20 data were analysed as scores (ranging from 0 to 60), as well as categorically to identify elevated depressive symptoms among those with scores of 16 or higher.^20 25^

### Statistical analyses

Sixty-two per cent (n=18 013) of the original baseline sample (n=28 945) participated in the 2006/8 follow-up investigation. Among those successfully re-examined, 14.3% (n=2575) were excluded due to missing study data, which resulted in an analytical sample of 15 438 participants (online supplementary figure S1). To account for depressive symptomatology at baseline in the prospective analyses, two strategies were undertaken. First, we fitted the three nested models (described below) on a sub-sample of participants with a CES-D 20 score <16 (n=10 372) at baseline. Second, we adjusted for baseline depressive symptoms by including CES-D 20 scores as a model 1 covariate on the entire analytical sample (n=15 438).

10.1136/jech-2018-211063.supp1 Supplementary file 1

The prospective association between social cohesion at baseline (using z-scores and tertiles) and elevated depressive symptoms (CES-D 10 score ≥4) at follow-up was examined using logistic regression. A statistically significant interaction was found between social cohesion and sex (p=0.016, test for interaction) but not by country and age. Hence, sex-specific nested models were estimated controlling for the following covariates: model 1 (age and country), model 2 (model 1 covariates plus marital status, educational level, economic deprivation and self-rated health) and model 3 which also adjusted for alcohol and smoking. We assessed the extent to which these behaviours changed the odds of having elevated depressive symptoms by social cohesion using the ‘difference method’ or the ‘proportion explained method’ to indirectly assess mediation.^26^

Before including the hypothesised mediators as covariates in the multivariable regression models, the properties of alcohol and smoking were considered. First, we discarded them as effect modifiers after finding no evidence of interaction between social cohesion tertiles and drinking frequency (p value for men=0.1403, p value for women=0.8017) and smoking status (p value for men=0.0937, p value for women=0.9242) on elevated depressive symptoms. We subsequently evaluated potential mediation using the Baron and Kenny procedures^27^ by verifying associations between cohesion tertiles and alcohol and smoking using multinomial logistic regression, and associations between each health behaviour and heightened symptoms using logistic regression.

We evaluated bias due to complete case analysis. We found statistically significant differences between complete and incomplete cases (online supplementary table S1), as the latter, for example, had lower socioeconomic positions, were less likely to be married/cohabiting and more likely to smoke. Additionally, they reported lower levels of cohesion, and greater elevated depressive symptoms at baseline and follow-up. These findings confirmed the data are not missing completely at random (MCAR). Where data are not MCAR, however, complete case analysis may provide unbiased estimates of the exposure OR under certain conditions. Briefly, the exposure OR is unbiased if the probability of being incomplete is independent of the outcome (Y), conditional on the exposure (X) and/or covariates (C).^28^ We tested these conditions by estimating whether the odds of being an incomplete case was dependent on Y, X and/or C included in each nested model. Being an incomplete case was not predicted by Y, conditional on X and covariates in any of the three nested models (online supplementary table S2); hence, we judged the exposure ORs among complete cases to be unbiased as per the methodological guidance.^28^

Analyses were conducted using Stata V.12.

## Results

Analytical sample characteristics by country and sex are shown in table 1. Overall, 31.4% of women compared with 17.9% of men experienced elevated depressive symptoms at follow-up. For both sexes, the prevalence of having a CES-D 10 score ≥4 appeared highest in Russians and lowest in Czech participants. Males and females had a mean baseline age of 58.2 and 57.7 years, respectively. Scores and tertiles of social cohesion were highest in Russia, followed by Poland and the Czech Republic. Men consistently reported higher alcohol drinking frequencies than women in all countries. Current smokers were more likely to be male in all countries, although this sex difference was most apparent in Russians. Most participants were either married or cohabiting, and well over half had attained a secondary or university level of education. Women in all countries appeared more economically deprived than men by a marginal degree. Men seemed more likely than women to report very good or good self-rated health. As during follow-up, baseline depressive symptoms appeared higher among women in all countries.

**Table 1:** Analytical sample characteristics by country and sex

Country	CZ (n=4677)		RU (n=4622)		PO (n=6139)		All (n=15 438)	
	Men	Women	Men	Women	Men	Women	Men	Women
Number of participants	2137	2540	2006	2616	2957	3182	7100	8338
**Follow-up measures (2006/2008)**								
Elevated depressive symptoms (CES-D 10 score >4) (%)	7.1	15.0	24.4	43.8	21.1	34.4	17.9	31.4
**Baseline measures (2002/2005)**								
Mean age (years)	58.8	57.9	57.9	58.0	58.0	57.7	58.2	57.7
Median social cohesion score (5–25)	15	15	16	17	16	16	16	16
Social cohesion tertiles (%)								
High (21–25)	27.9	27.6	49.7	50.8	48.5	43.2	40.5	40.9
Medium (18–20)	37.4	35.8	27.4	27.9	33.8	32.1	33.0	31.9
Low (5–17)	34.7	36.7	22.9	21.3	22.8	24.6	26.4	27.5
Drinking frequency (%)								
Never	4.9	13.7	12.7	15.1	19.9	43.2	13.4	25.5
Less than once a month	16.3	33.5	16.8	54.7	19.8	27.2	17.9	37.8
One to three times a month	17.9	25.4	22.4	21.4	23.5	18.7	21.5	21.6
One to four times a week	37.5	22.8	43.3	8.6	29.5	10.0	35.8	13.4
Five or more times a week	23.3	4.6	4.8	0.3	7.3	0.9	11.4	1.8
Smoking status (%)								
Never	34.6	56.5	26.1	86.4	29.9	51.3	30.2	63.9
Past	38.2	21.8	27.8	4.9	36.3	21.3	34.5	16.3
Current	27.2	21.7	46.1	8.7	33.8	27.5	35.3	19.8
Married or cohabiting (%)	85.3	69.6	89.3	61.6	89.0	68.3	88.0	66.6
Educational level (%)								
Primary or less	4.2	14.4	4.7	5.0	8.0	11.8	5.9	10.4
Vocational	40.5	28.9	22.9	30.6	26.0	15.2	29.5	24.2
Secondary	34.3	44.5	36.5	35.4	33.6	43.9	34.6	41.4
University	21.1	12.2	35.9	29.0	32.4	29.1	30.0	23.9
Median economic deprivation score (0–12)	0	1	2	4	0	1	0	2
Self-rated health (%)								
Very good	3.0	3.7	0.2	0.1	4.9	3.2	3.0	2.4
Good	40.5	41.3	16.0	5.2	37.2	30.6	32.2	25.9
Fair	47.5	46.2	69.9	68.7	45.5	52.5	53.0	55.7
Poor	8.4	8.4	13.5	24.3	11.4	12.5	11.1	14.9
Very poor	0.7	0.4	0.5	1.8	1.1	1.2	0.8	1.2
Median CES-D 20 score (0–60)	7	9	9	12	8	11	8	11
Elevated depressive symptoms (CES-D 20 score ≥16) (%)	11.1	21.5	14.5	32.5	18.7	30.2	15.3	28.2

*CES-D, Center for Epidemiological Depression; CZ, Czech Republic; PO, Poland; RU, Russia.

Table 2 reports the sex-specific associations between baseline social cohesion and elevated depressive symptoms at follow-up, among participants without heightened symptoms at baseline. Controlling for age and country, the risk of having a CES-D 10 score ≥4 increased as social cohesion levels decreased in both men and women (model 1). This effect was greater in men than in women. For every SD decrease in the social cohesion z-score, the odds of having elevated depressive symptoms increased by 17% (OR=1.17, 95% CI 1.08 to 1.26) in men, compared with 10% (OR=1.10, 95% CI 1.03 to 1.17) in women. Consistently, gradients were found in the odds of elevated depressive symptoms from high to low tertiles of social cohesion, but this trend was stronger in men.

**Table 2:** Sex-specific ORs of elevated depressive symptoms (CES-D 10 score ≥4) at follow-up by social cohesion at baseline among participants with a CES-D 20 baseline score <16

	Model 1*		Model 2†		Model 3‡	
	OR	95% CI	OR	95% CI	OR	95% CI
Men						
Per 1 SD decrease	1.17	1.08 to 1.26	1.13	1.05 to 1.23	1.13	1.05 to 1.22
High tertile	Reference		Reference		Reference	
Medium tertile	1.33	1.12 to 1.58	1.28	1.07 to 1.53	1.28	1.07 to 1.53
Low tertile	1.42	1.18 to 1.72	1.34	1.10 to 1.63	1.33	1.10 to 1.62
Women						
Per 1 SD decrease	1.10	1.03 to 1.17	1.05	0.99 to 1.13	1.05	0.99 to 1.13
High tertile	Reference		Reference		Reference	
Medium tertile	1.23	1.07 to 1.42	1.20	1.04 to 1.38	1.20	1.04 to 1.38
Low tertile	1.28	1.10 to 1.50	1.18	1.01 to 1.39	1.18	1.01 to 1.39

*Adjusted for age and country.

†Adjusted for model 1 covariates plus marital status, educational level, economic deprivation and self-rated health.

‡Adjusted for model 2 covariates plus drinking frequency and smoking status.

CES-D, Center for Epidemiological Depression.

Further adjustment for socioeconomic factors and self-rated health (model 2) weakened associations in both sexes, but they remained statistically significant. For instance, the risk of having high depressive symptoms increased by 13% (OR=1.13, 95% CI 1.05 to 1.23) in men and 5% (OR=1.05, 95% CI 0.99 to 1.13) in women for 1 SD decrease in social cohesion z-score. The trend across social cohesion tertiles remained stepwise for men as odds increased from the medium (OR=1.28, 95% CI 1.07 to 1.53) to the low (OR=1.34, 95% CI 1.10 to 1.63) tertile, but the graded association disappeared for women as the higher odds for the medium and low tertiles were broadly equivalent at 20% and 18%. Lastly, model 3 showed that the odds of having elevated depressive symptoms by social cohesion z-scores or tertiles were not affected by drinking frequency and smoking status.

The alternative analysis, which adjusted for baseline depressive symptoms (table 3), also found stronger effects in men, and no indirect evidence that drinking frequency and smoking status played a role in the prospective association between social cohesion and heightened depressive symptoms.

**Table 3:** Sex-specific ORs of elevated depressive symptoms (CES-D 10 score ≥4) at follow-up by social cohesion at baseline

	Model 1*		Model 2†		Model 3‡	
	OR	95% CI	OR	95% CI	OR	95% CI
Men						
Per 1 SD decrease	1.19	1.11 to 1.27	1.15	1.07 to 1.23	1.15	1.07 to 1.23
High tertile	Reference		Reference		Reference	
Medium tertile	1.33	1.14 to 1.56	1.28	1.09 to 1.50	1.28	1.09 to 1.50
Low tertile	1.47	1.24 to 1.73	1.37	1.16 to 1.62	1.38	1.16 to 1.63
Women						
Per 1 SD decrease	1.09	1.04 to 1.15	1.06	1.00 to 1.11	1.06	1.00 to 1.11
High tertile	Reference		Reference		Reference	
Medium tertile	1.11	0.99 to 1.25	1.08	0.96 to 1.22	1.08	0.96 to 1.22
Low tertile	1.31	1.15 to 1.49	1.22	1.07 to 1.39	1.22	1.07 to 1.39

*Adjusted for age, country and CES-D 20 score at baseline.

†Adjusted for model 1 covariates plus marital status, educational level, economic deprivation and self-rated health.

‡Adjusted for model 2 covariates plus drinking frequency and smoking status.

CES-D, Center for Epidemiological Depression.

## Discussion

This study suggested that social cohesion had a protective role on the risk of experiencing high depressive symptomatology after a 3-year period among older urban-dwelling adults in the Czech Republic, Russia and Poland. A dose-response relationship was observed whereby the risk of elevated depressive symptoms escalated with decreasing levels of social cohesion, although this was stronger in men than in women. Although theory has suggested that this relationship may operate through a health behavioural pathway, as cohesion may regulate behavioural norms and protect against daily stressors related to health-damaging behaviours^5 29^; our study found that alcohol and smoking played no appreciable role in this relationship.

To our knowledge, this is the first longitudinal analysis exploring the association between social cohesion—or other markers of cognitive social capital—and depressive symptoms in CEE. Despite the paucity of evidence from this region,^8–10^ cross-sectional associations were found between interpersonal trust and psychological distress in nine former Soviet Union countries,^30 31^ but not with subjective well-being in older Polish adults.^32^ Perceived safety was associated with fewer depressive symptoms in Ukrainian women, but no cross-sectional associations were observed in Ukrainian men.^33^ Our study on urban-dwelling older adults in Czech Republic, Russia and Poland limit the generalisability of our findings for the region, but coincide with prospective findings on older adults from other regions. Social cohesion, interpersonal trust and reciprocity predicted fewer depressive symptoms over follow-up in ageing populations from England,^34^ Japan^35^ and Korea,^36^ but not from the USA.^37 38^

The stronger associations in CEE men is contrary to findings by Karhina *et al* who found that perceived safety was only protective against depressive symptoms in Ukrainian women.^33^ Although empirical evidence from CEE is lacking; it is generally accepted that women have more emotionally intimate relationships, and actively draw on social support during stressful periods, in comparison to men.^29^ Hence, lower social cohesion may be more harmful for older CEE men, because they lack the social and emotional resources that women rely on to offset the harms associated with low social cohesion.

Conceptual frameworks on the psychosocial environment and mental health suggest that health behaviours are a potential pathway by which social cohesion can influence depressive disorders, but this mechanism has been largely overlooked in the literature.^5 29^ As drinking frequency and smoking status did not explain the inverse relationship between lower levels of social cohesion and higher odds of elevated depressive symptoms after controlling for confounders, the unexplained associations in our data suggest that social, psychological and physiological pathways may be at play.^5 29^

Several limitations must be acknowledged. First, the prospective (although short-term) nature of this study may have insufficiently addressed reverse causality because depressed cases may be prone to cognitive distortions,^39^ such as negatively perceiving interpersonal relations that embody social cohesion. While the prospective analyses accounted for baseline risk of depression, this phenomenon may partly influence our findings. Second, the potentially long-term effect of social cohesion could not be explored as no residential history/mobility data were available for analysis. Relatedly, social cohesion levels may have varied over the 3-year period, but this study could not assess changes as cohesion data were collected only at baseline. Third, although we controlled for key socioeconomic characteristics, the observed association may be confounded by unmeasured aspects of SEP, such as occupational status and income.

Moreover, comparison of the difference between unadjusted and adjusted estimates to explore mediation may be regarded as too crude compared with more advanced methods. While our chosen approach has been regarded as a conservative evaluation of mediation, it can establish the presence of a potential mediating effect where it occurs.^26^ As the inclusion of alcohol and smoking did not change estimated ORs by more than a value of 0.01, more advanced techniques were not subsequently undertaken. Since advanced methods recommend a longitudinal study design, this may not have been appropriate in our study as the exposure and mediators were measured concurrently. Hence, our study cannot discard plausible cause and effect from health behaviours to social cohesion.

While loss to follow-up reduced the representativeness of the analytical sample, and complete case analysis can be biased in particular circumstances, our model-specific diagnostic checks showed no evidence that our reported findings were partial to these conditions. We therefore conclude that complete case analysis only resulted in a loss of statistical power, an ignorable issue given our large analytical sample. Measuring caseness according to self-reported depressive symptoms, and not on clinical diagnosis, may misclassify participants with transient symptoms or less severe affect states as having major depression. However, the CES-D threshold has been highly prognostic of clinical diagnoses in older populations.^20 40^ Different versions of the CES-D were employed at baseline and follow-up. While this may be considered as a further limitation, our aim was not to compare symptom changes between the two time points.

Although the role of cognitive social capital, including social cohesion, on the risk of depressive disorders has been understudied in CEE and limited to cross-sectional evidence, our study found strong prospective associations among older Czech, Russian and Polish adults. Our work highlights the importance of additional evidence on the role of the psychosocial environment on mental health in these populations.

## Conclusions

Lower levels of social cohesion predicted elevated depressive symptoms after a 3-year follow-up among older Czech, Russian and Polish adults. The association appeared stronger in men, and there was no evidence of mediation by drinking and smoking.

**What is already known on this subject**

- A protective effect of cognitive social capital on depressive disorders is supported by an increasing body of evidence, but this is limited to particular cultural settings, such as the USA and Northern/Western Europe.
- Whether these associations exist in under-represented regions, such as Central and Eastern Europe has yet to be explored.

**What this study adds**

- Low social cohesion is associated with a higher risk of elevated depressive symptoms among adults in three Central and Eastern European countries.
- Health behaviours do not seem to explain this association; therefore, further research should elucidate the underlying pathways between cognitive social capital and mental health disorders in these populations.
