# Supplementary material for: The prospective relationship between social cohesion and depressive symptoms among older adults from Central and Eastern Europe
Source: J Epidemiol Community Health. 2018 Nov 1;73(2):117–22. doi: 10.1136/jech-2018-211063 (PMC6352418; doi:10.1136/jech-2018-211063)
Supplement: Supplementary file 1 [file jech-2018-211063supp001.pdf]

Title: The prospective relationship between social cohesion and depressive symptoms among older adults from Central and Eastern Europe

List of Authors: Carla Bertossi Urzua*[1], Milagros A Ruiz*^[1], Andrzej Pajak[2], Magdalena Kozela[2], Ruzena Kubinova[3], Sofia Malyutina[4,5], Anne Peasey[1], Hynek Pikhart[1], Michael Marmot[1,6], Martin Bobak[1]
**Joint first authors*

Author Affiliations: [1]Research Department of Epidemiology and Public Health, University College London, London, United Kingdom; [2]Institute of Public Health, Faculty of Health Sciences, Jagiellonian University Medical College, Krakow, Poland; [3]Centre for Environmental Health Monitoring, National Institute of Public Health, Prague, Czech Republic; [4]Research Institute of Internal and Preventive Medicine, Branch of the Institute of Cytology and Genetics, SB RAS, Novosibirsk, Russia; [5]Novosibirsk State Medical University, Novosibirsk, Russia; [6]UCL Institute of Health Equity and Research Department of Epidemiology and Public Health, University College London, London, United Kingdom

^Correspondence to m.a.ruiz@ucl.ac.uk

Supplementary Figure and Tables

Figure S1 Selection diagram of analytic sample 2

Table S1 Analytic sample characteristics between complete (N=15,438) and incomplete cases due to missing data (n=2,584) or loss to follow-up (n=10,932) 3

Table S2 Odds ratios (ORs) of being an incomplete case due to missing data or loss to follow-up by analysis variables 4

Figure S1 Selection diagram of analytic sample

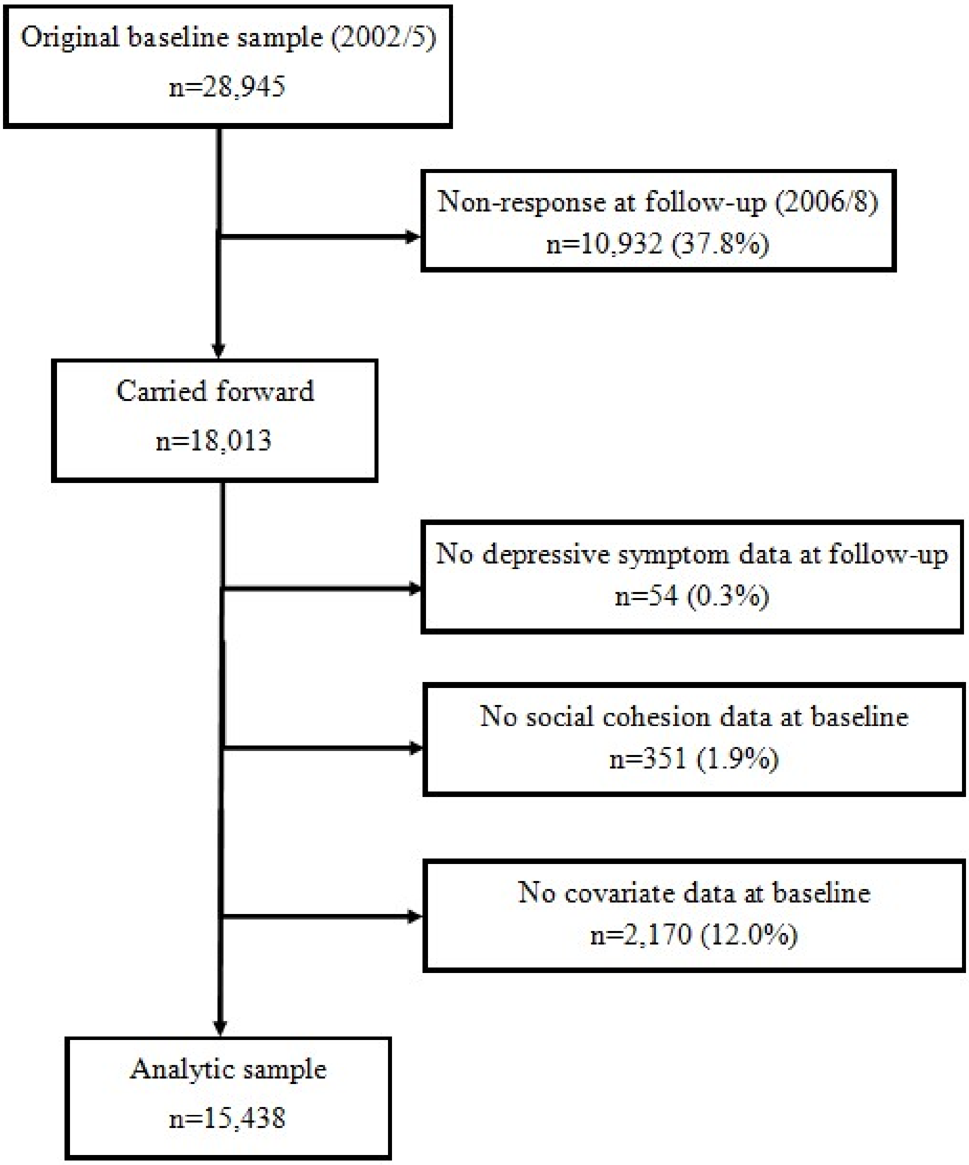

Table S1 Analytic sample characteristics between complete (N=15,438) and incomplete cases due to missing data (n=2,584) or loss to follow-up (n=10,932)

Study data*	Complete cases (N=15,438)	Incomplete cases (N=13,516)	P-value
**Follow-up measures (2006/8)**			
Elevated depressive symptoms (CES-D 10 score ≥ 4) (%)	25.2	33.9	<0.001
**Baseline measures (2002/5)**			
Mean age (years)*	57.9	58.1	0.0212
Female (%)*	54.0	51.7	<0.001
Country (%)*			
Czech Republic	30.2	31.0	
Russia	30.0	35.0	
Poland	39.8	34.0	<0.001
Median social cohesion score (5-25)	20	20	<0.001
Social cohesion tertiles (%)			
High (21-25)	40.7	36.7	
Medium (18-20)	32.4	32.8	
Low (5-17)	26.9	30.5	<0.001
Drinking frequency (%)			
Never	19.9	24.3	
Less than once a month	28.6	29.1	
One to three times a month	21.6	19.4	
One to four times a week	23.7	21.1	
Five or more times a week	6.2	6.1	<0.001
Smoking status (%)			
Never	48.6	45.2	
Past	24.7	23.0	
Current	26.7	31.9	<0.001
Married or cohabitating (%)	76.4	72.9	<0.001
Educational level (%)			
Primary or less	8.3	15.2	
Vocational	26.6	29.2	
Secondary	38.3	34.5	
University	26.8	21.2	<0.001
Median deprivation score (0-12)	1	2	<0.001
Self-rated health (%)			
Very good	2.7	2.3	
Good	28.8	23.5	
Fair	54.4	55.4	
Poor	13.2	17.1	
Very Poor	1.0	1.8	<0.001
Median CES-D 20 score (0-60)	0	10	<0.001
Elevated depressive symptoms (CES-D 20 score ≥ 16) (%)	22.3	26.6	<0.001

* Covariate baseline data on age, sex and country were observed for all participants.

Table S2 Odds ratios (ORs) of being an incomplete case due to missing data or loss to follow-up by analysis variables

	Model 1			Model 2			Model 3		
	OR	95% CI	P-value	OR	95% CI	P-value	OR	95% CI	P-value
**Y**									
Elevated depressive symptoms (CES-D 10 score ≥ 4)	0.79	0.60, 1.04	0.093	0.82	0.57, 1.20	0.313	0.83	0.54, 1.29	0.417
**X**									
Social cohesion z-score	0.88	0.79, 0.98	0.024	0.90	0.77, 1.04	0.150	1.14	0.98, 1.04	0.157
**C**									
Age*	1.02	1.01, 1.04	0.001	1.02	1.00, 1.04	0.078	1.01	0.98, 1.04	0.437
Female*	1.11	1.23, 1.71	0.329	1.49	1.09, 2.04	0.011	1.70	1.11, 2.60	0.015
Country*	0.81	0.71, 0.92	0.001	0.79	0.66, 0.94	0.008	0.86	0.69, 1.07	0.170
CES-D 20 score (0-60)	1.00	0.99, 1.01	0.857	1.00	0.99, 1.03	0.541	1.01	0.99, 1.03	0.268
Married or cohabitating	-	-	-	0.77	0.56, 1.07	0.120	0.66	0.45, 0.96	0.028
Educational level	-	-	-	1.18	1.01, 1.39	0.034	1.01	0.83, 1.22	0.935
Deprivation score	-	-	-	0.99	0.94, 1.05	0.700	0.97	0.91, 1.04	0.391
Self-rated health	-	-	-	0.76	0.61, 0.94	0.013	0.68	0.52, 0.88	0.004
Drinking frequency	-	-	-	-	-	-	0.76	0.64, 0.91	0.002
Smoking status	-	-	-	-	-	-	1.00	0.80, 1.24	0.977

* Covariate was observed for all participants.
